# Quasi Single Point Calibration Method for High-Speed Measurements of Resistive Sensors

**DOI:** 10.3390/mi10100664

**Published:** 2019-09-30

**Authors:** Jesús A. Botín-Córdoba, Óscar Oballe-Peinado, José A. Sánchez-Durán, José A. Hidalgo-López

**Affiliations:** 1 Departamento de Electrónica, Universidad de Málaga, Andalucía Tech, Campus de Teatinos, 29071 Málaga, Spain; jesus.botin@uma.es (J.A.B.-C.); oballe@uma.es (Ó.O.-P.); jsd@uma.es (J.A.S.-D.); 2 Instituto de Investigación Biomédica de Málaga (IBIMA), 29010 Málaga, Spain

**Keywords:** direct interface circuits, calibration methods, error analysis, resistive tactile sensor, time-based measurement

## Abstract

Direct interface circuits are a simple, inexpensive alternative for the digital conversion of a sensor reading, and in some of these circuits only passive calibration elements are required in order to carry out this conversion. In the case of resistive sensors, the most accurate methods of calibration, namely two-point calibration method (TPCM) and fast calibration methods I and II (FCMs I and II), require two calibration resistors to estimate the value of a sensor. However, although FCMs I and II considerably reduce the time necessary to estimate the value of the sensor, this may still be excessive in certain applications, such as when making repetitive readings of a sensor or readings of a large series of sensors. For these situations, this paper proposes a series of calibration methods that decrease the mean estimation time. Some of the proposed methods (quasi single-point calibration methods) are based on the TPCM, while others (fast quasi single-point calibration methods) make the most of the advantages of FCM. In general, the proposed methods significantly reduce estimation times in exchange for a small increase in errors. To validate the proposal, a circuit with a Xilinx XC3S50AN-4TQG144C FPGA has been designed and resistors in the range (267.56 Ω, 7464.5 Ω) have been measured. For 20 repetitive measurements, the proposed methods achieve time reductions of up to 61% with a relative error increase of only 0.1%.

## 1. Introduction

Sensors interfacing with digital devices may be one of the most popular topics in electronics today. We can find a multitude of very different applications where this type of circuit plays a fundamental role, such as checking on patients [1], soil pore-water salinity sensors [2], monitoring composting processes [3], or specially piezoresistive tactile sensors [4]. Direct interface circuits (DICs) [5] are a series of circuits based on methods that use a small number of additional components to make the connection between a sensor and a programmable digital device (PDD). There are DICs for resistive sensors [6,7,8,9,10,11,12,13,14], capacitive [15,16,17] or inductive [18,19,20,21,22] sensors, and even DICs built to measure any of them [23].

In some cases, the DICs use, together with the sensor, only additional passive components. In other cases, the additional components may be a transistor or even a logic gate. Another important difference between the various types of DICs is the use of analogue elements that may be included in the PDDs. For example, we currently find microprocessors that can include analogue-to-digital converters (ADCs) or analogue comparators. With performances being equal, DICs that do not require additional active components or analogue elements within the PDDs will obviously be preferred. This type of DIC*s* usually performs a time-to-digital conversion in the PDD in order to obtain the value of the physical magnitude to be measured and will be used in this paper for the specific case of resistive sensors. Using a DIC can result in a reduction in costs, complexity, and power consumption in the measurement chain compared to the use of a traditional scheme with ADCs and signal conditioning circuits.

DIC parameters such as uncertainties, effective numbers of bits, calibrations, resolution, or response to dynamic signals have already been well explored and analyzed [9,22,24,25,26,27,28]. However, the time needed to perform the conversion has received little attention when this parameter is in fact one of the most important characteristics for sensors, given the need for data acquisition speeds. This is crucial in the case of DICs for resistive sensors based on time-to-digital conversion since the sensor’s resistance value range can be very broad, meaning an extensive time range is necessary for conversion. Furthermore, in order to improve accuracy in estimating the resistance value of the sensor, *R*, it is necessary to carry out additional readings of certain calibration resistors, therefore increasing the total time necessary to obtain *R*. This problem is increased when reading groups or arrays of resistive sensors, as usually happens in applications with tactile sensors or electronic skins.

The aim of the calibration resistors is to eliminate the influence that different parameters have on accuracy in estimating *R*. Apart from the internal resistance of the buffers of the pins of the PDDs, *Ro*, other factors that introduce errors in the measurement are the variability in: the capacitance used in the estimation process, *C*, the value in the supply voltage, *V_DD_*, and the threshold voltage of the pins of the PDDs, *V_f_*. All these possible sources of error can be compensated thanks to calibration resistors. Another possible source of error would be the self-heating of the resistors. However, this phenomenon can be neglected in DICs, since the current does not pass through the resistors continuously and its value is low due to the high resistance normally shown by resistive sensors.

Since calibration resistors must also increase their value in line with the increase in the maximum resistance value to be measured [26], the total time needed to estimate *R*, *T_E_(R)*, can result in a high value. If the resistance value of a sensor is only to be measured sporadically, this problem may not be particularly important. However, if repetitive measurements are to be made for a single sensor or information can be obtained for different sensors (or even if these two situations occur simultaneously), then *T_E_(R)* becomes a fundamental parameter that can considerably slow down an application, or, in certain cases, even prevent the use of DIC*s*. Finding a method to reduce *T_E_(R)* can be crucial in some practical applications, such as tactile sensors, where the number of resistive sensors to be read can be very high and, moreover, a high reading frequency of the sensors is required in order to calculate characteristics such as grips or slippages [29,30].

This paper presents a method for estimating *R* which allows *T_E_(R)* to be reduced without the need for additional hardware while maintaining adequate accuracy in the measurement, making it possible to use DICs in applications such as those indicated above.

The structure of the paper is as follows. In Section 2 different calibrations methods are evaluated regarding accuracy and time needed to estimate *R*. Section 3 presents two new calibration methods to increase the speed of a DIC. In Section 4, experimental results and discussion about these new methods are performed. Finally, Section 5 presents the main conclusions.

## 2. Evaluating *R* Estimation Time in Classical Calibration Methods

**Figure 1:**
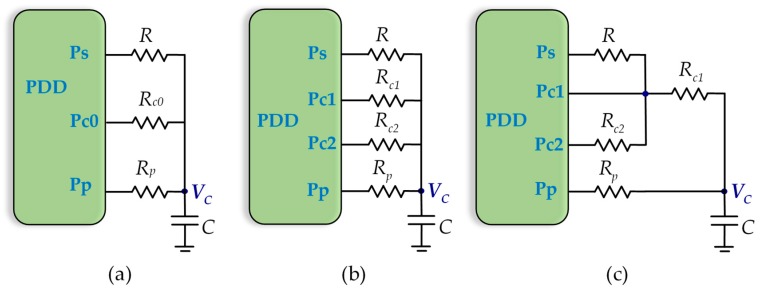
Different types of direct interface circuits (DICs): (**a**) single-point calibration method (SPCM); (**b**) two-point calibration method (TPCM); (**c**) three-signal calibration method (TSCM).

There are three classic calibration methods for a DIC, differing in the number of calibration resistors and how these resistors are used to discharge the capacitor. The circuits used in each of the methods are shown in Figure 1. Figure 1a shows the circuit for the single-point calibration method (SPCM). In this circuit, a capacitor is first charged to *V_DD_* through the Pp pin of the PDD (resistor *Rp* is optional and only necessary if the current through the pin needs to be limited, although in the literature we can also find that this resistor can reduce the influence of the power-supply noise [31]). After charging, a discharge is made either through *R* or through a calibration resistor, *R_c0_*. This is done by configuring the pin connected to the resistor that discharges as a logic 0 output, while the pin of the other resistor is configured as a high-impedance output. This way of proceeding in the discharge of a resistor will be called the normal discharge procedure. The discharge ends when the Pp pin (configured as the high-impedance input during discharge) detects a change to the logic 0 input. The capacitor is then re-charged and finally discharged again through the other resistor until a new logic 0 input is detected.

This method, taking into account the discharge equations of a capacitor, obtains [5]:

(1) $$\frac{T_{R}}{T_{Rc0}}=\frac{\left(R+R_{o}\right)C\ln\frac{V_{DD}}{V_{f}}}{\left(R_{Rc0}+R_{o}\right)C\ln\frac{V_{DD}}{V_{f}}}=\frac{R+R_{o}}{R_{c0}+R_{o}}$$

where *T_R_* and *T_Rc0_* are the discharge times from *V_DD_* to *V_f_* through *R* and *R_c0_*, respectively (the sub-index of the time measurement will always indicate which resistor it is discharged through). These times will be expressed as the number of PDD clock cycles occurring during the discharge process. Since *R_o_* is unknown, we cannot obtain the value of *R* based on Equation (1). However, if, as usually occurs, *R_o_* is a small value compared to those of the different resistors to be measured, it is possible to approximate:

(2) $$R\approx \frac{T_{R}}{T_{Rc0}}R_{c0}$$

Improving estimation of *R* provided by Equation (2) and eliminating error due to *R_o_* requires methods and circuits that use two calibration resistors: two-point calibration method (TPCM) and three-signal calibration method (TSCM) [5]. The TPCM, as shown in Figure 1b, carries out the same processes as the SPCM, but with two calibration resistors, *R_c1_* and *R_c2_*. In this method, the calculation of *R* is given [5] by:

(3) $$R=\frac{T_{R}-T_{Rc1}}{T_{Rc2}-T_{Rc1}}\left(R_{c2}-R_{c1}\right)+R_{c1}$$

For its part, the TSCM, as shown in Figure 1c, performs three discharge processes, like the TPCM, but through *R_c1_*, *R*+*R_c1_*, and *R_c2_*+*R_c1_*. Proceeding in this way makes the equation for determining *R* simpler [32], which can be written as:

(4) $$R=\frac{T_{R+Rc1}-T_{Rc1}}{T_{Rc2+Rc1}-T_{Rc1}}R_{c2}$$

*T_E_(R)* is different for each of the aforementioned methods. If we define *T_charge_* as the time necessary for the capacitor charge, the values of *T_E_(R)* for the different methods are given by:

(5) $$T_{E}(R,SPCM)=2T_{\mathrm{charge}}+T_{R}+T_{Rc0}$$

(6) $$T_{E}(R,TPCM)=3T_{\mathrm{charge}}+T_{R}+T_{Rc1}+T_{Rc2}$$

(7) $$T_{E}(R,TSCM)=3T_{\mathrm{charge}}+T_{R+Rc1}+T_{Rc2+Rc1}+T_{Rc1}$$

where the second term in parenthesis for *T_E_* indicates which calibration method it is calculated with.

Two aspects must be considered with regard to Equations (5)–(7). Firstly, *T_charge_* is much lower than the other times of these equations, since, as mentioned, *R_p_* is either very small or not necessary, meaning we can eliminate this term from these equations. Secondly, calibration resistors are not the same in all methods. A common choice for the SPCM is to place *R_c0_* in the middle of the range of resistors to be measured [*R_min_, R_max_*], in order to minimize the maximum error when using Equation (2). Given this, Equation (5) becomes:

(8) $$T_{E}(R,SPCM)\approx T_{R}+T_{\frac{R\max+R\min}{2}}\approx T_{R}+\frac{T_{R\max}+T_{R\min}}{2}$$

where we have taken into consideration that *R_o_* is very small compared to those of the resistors to be measured.

For its part, optimal performance in the TPCM requires *R_c1_* and *R_c2_* to be, respectively, in 15% and 85% of the resistance value range to be measured in order to minimize errors in estimation [26], meaning Equation (6) can be written as:

(9) $$T_{E}(R,TPCM)\approx T_{R}+\underset{T_{Rc1}}{\underset{︸}{0.15\cdot T_{R\max}+0.85\cdot T_{R\min}}}+\underset{T_{Rc2}}{\underset{︸}{0.85\cdot T_{R\max}+0.15\cdot T_{R\min}}}=T_{R}+T_{R\max}+T_{R\min}$$

The literature does not describe any criteria for choosing *R_c1_* and *R_c2_* in the TSCM, and, although it is at least possible to maintain the 85% criterion for *R_c2_*, it is obvious that *R_c1_* cannot be in 15% of the range as its minimum value is *R_min_* +*R_c1_*. The systematic error made by this method will therefore always be greater than that obtained by the TPCM [33]. In any case, if we maintain the same criteria of 15% and 85% to situate the calibration resistors, we will have:

(10) $$\begin{array}{c} T_{E}(R,TSCM)\approx \underset{T_{R+Rc1}}{\underset{︸}{T_{R}+0.15\cdot T_{R\max}+0.85\cdot T_{R\min}}}+\underset{T_{Rc1}}{\underset{︸}{0.15\cdot T_{R\max}+0.85\cdot T_{R\min}}}+\underset{T_{Rc2}+T_{Rc1}}{\underset{︸}{0.85\cdot T_{R\max}+0.15\cdot T_{R\min}}}=\\ =T_{R}+1.15\cdot T_{R\max}+1.85\cdot T_{R\min} \end{array}$$

Comparing Equations (9) and (10), it is obvious that *T_E_(R,*TSCM*)* > *T_E_(R,*TPCM*),* meaning that, in terms of temporal performances for the same accuracy, the TPCM outperforms the TSCM [33]. It is also obvious that *T_E_(R,*SPCM*)* < *T_E_(R,*TPCM*),* although the TPCM is more accurate and thus, the application for which the DIC is used will determine which one to use. Given the foregoing, the ideal situation would be to find a calibration method with a *T_E_(R)* similar to or lower than *T_E_(R,*SPCM*)* and an accuracy equivalent to that obtained by the TPCM. The following section presents two new calibration methods that meet these two requirements when measuring the resistance value of a large number of sensors using the same DIC or carrying out repetitive measurements of the same sensor. We call these new methods quasi single-point calibration methods (QSPCMs).

## 3. Quasi Single-Point Calibration Methods

### 3.1. Quasi Single-Point Calibration Method

Let us suppose that, as in the TPCM, we want to use two calibration resistors in order to repetitively obtain the resistance value of a sensor (with the aim of achieving the maximum accuracy). Let $T_{R}^{n}$, $T_{Rc1}^{n}$ and $T_{Rc2}^{n}$ be the discharge times to obtain the *n*^th^ estimation of *R*, *R^n^* (the superscript will be used to indicate the estimation number in all variables). Since the calibration resistors are fixed, we have the following relationship if Equation (3) is used for an initial estimation of *R*:

(11) $$\frac{T_{R}^{0}-T_{Rc1}^{0}}{T_{Rc2}^{0}-T_{Rc1}^{0}}=\frac{\left(R^{0}+R_{o}^{0}\right)C\ln\frac{V_{VDD}^{0}}{V_{f}^{0}}-\left(R_{c1}+R_{o}^{0}\right)C\ln\frac{V_{VDD}^{0}}{V_{f}^{0}}}{\left(R_{c2}+R_{o}^{0}\right)C\ln\frac{V_{VDD}^{0}}{V_{f}^{0}}-\left(R_{c1}+R_{o}^{0}\right)C\ln\frac{V_{VDD}^{0}}{V_{f}^{0}}}=\frac{R^{0}-R_{c1}}{R_{c2}-R_{c1}}$$

It should be remembered that in Equation (11) *V_DD_*, *V_f_*, and *R* are considered to be the same for the three measurements, since the temporal moments of the discharges of the series of measurements for an estimation are very close to each other (this approximation is one of the sources of error in any type of DIC). $T_{R}^{n}$, $T_{Rc1}^{n}$, and $T_{Rc2}^{n}$ are measured again in any new estimation of *R* and *R^n^*, thus updating the values of the voltages and *R_o_*.

However, if the aim is to speed up the estimation of *R^n^*, we can also use the data from the initial estimation of *R* as follows:

(12) $$\begin{array}{c} \frac{T_{R}^{n}-T_{Rc1}^{n}}{T_{Rc2}^{0}-T_{Rc1}^{0}}\cdot \frac{T_{Rc1}^{0}}{T_{Rc1}^{n}}=\frac{\left(R^{n}+R_{o}^{n}\right)C\ln\frac{V_{VDD}^{n}}{V_{f}^{n}}-\left(R_{c1}+R_{o}^{n}\right)C\ln\frac{V_{VDD}^{n}}{V_{f}^{n}}}{\left(R_{c2}+R_{o}^{o}\right)C\ln\frac{V_{VDD}^{0}}{V_{f}^{0}}-\left(R_{c1}+R_{o}^{o}\right)C\ln\frac{V_{VDD}^{0}}{V_{f}^{0}}}\cdot \frac{\left(R_{c1}+R_{o}^{o}\right)C\ln\frac{V_{VDD}^{0}}{V_{f}^{0}}}{\left(R_{c1}+R_{o}^{n}\right)C\ln\frac{V_{VDD}^{n}}{V_{f}^{n}}}=\\ =\frac{R^{n}-R_{c1}}{R_{c2}-R_{c1}}\cdot \frac{R_{c1}+R_{o}^{o}}{R_{c1}+R_{o}^{n}} \end{array}$$

With Equation (12), we can solve *R^n^*:

(13) $$R^{n}=\frac{R_{c1}+R_{o}^{n}}{R_{c1}+R_{o}^{0}}\cdot \frac{T_{R}^{n}-T_{Rc1}^{n}}{T_{Rc2}^{0}-T_{Rc1}^{0}}\cdot \frac{T_{Rc1}^{0}}{T_{Rc1}^{n}}\cdot \left(R_{c2}-R_{c1}\right)+R_{c1}$$

The first quotient of the member on the right of this equation is a term very close to one, since *R_o_* << *R_c1_,* and, moreover, the variations of *R_o_* over time (due to the circuit conditions) are even smaller, meaning $R_{o}^{0}\approx R_{o}^{n}$. Hence, if we define:

(14) $$A=\frac{T_{Rc1}^{0}}{T_{Rc2}^{0}-T_{Rc1}^{0}}$$

based on Equation (13), we can write Equation (15) that describes the QSPCM:

(15) $$R^{n}=\frac{T_{R}^{n}-T_{Rc1}^{n}}{T_{Rc1}^{n}}A\left(R_{c2}-R_{c1}\right)+R_{c1}$$

According to the method determined by Equation (15), when estimating *R*^0^ we will need to evaluate the discharges through *R_c1_* and *R_c2_*; for any other estimate of *R*, we only need to evaluate the discharge through *R_c1_*. Equation (15) can be applied not only for a succession of measurements of the same sensor, but also when a series of resistive sensors is being measured (in this situation, *R^n^* would be the *n*^th^ resistive sensor of the series).

As the value of *R_c2_* is close to the highest value to be measured and its discharge time is only necessary in evaluating *R*^0^, it is significantly time-saving in the estimations of *R^n^*. Moreover, it is obvious that the hardware necessary to use Equation (15) in a DIC is the same as for the TPCM, meaning there is no additional hardware cost. There may only be a computational cost in estimating *R*^0^ derived from a quotient and an added multiplication in Equation (15) compared to with Equation (3). However, if the product $A\left(R_{c2}-R_{c1}\right)$ is stored in the PDD memory, starting from *R*^1^, the number of arithmetic operations is lower in the QSPCM than in the TPCM, since subtraction of the denominator of the quotient is removed. 

The main drawback in using the QSPCM is the small increase in error due to use of the approximation that eliminates the first quotient of the term on the right side of Equation (13). This phenomenon is studied in Section 4.

The QSPCM allows for an additional reduction in the uncertainty of the estimation of *R* if a mean of the discharge times of the calibration resistors of the first *j* measurement cycles is used instead of $T_{Rc1}^{0}$ and $T_{Rc2}^{0}$. This mean value, $\mu_{j}(T_{Rc})$, is defined by:

(16) $$\mu_{j}(T_{Rc})=\frac{1}{j}\sum_{i=0}^{j-1}T_{Rc}^{i}$$

where 1 < *j* << *n* is necessary in order to achieve significant time-savings in the measurements. We can also define a new *A ^j^*, similar to *A* that appears in Equation (14), taking these means into account:

(17) $$A^{j}=\frac{\mu_{j}(T_{Rc1})}{\mu_{j}(T_{Rc2})-\mu_{j}(T_{Rc1})}$$

Proceeding in this way, we obtain Equation (18) equivalent to Equation (15):

(18) $$R^{n}=\frac{T_{R}^{n}-T_{Rc1}^{n}}{T_{Rc1}^{n}}A^{j}\left(R_{c2}-R_{c1}\right)+R_{c1},\;1<j\ll n$$

Equation (18) presents a lower uncertainty in estimating *R* and only a small increase in the temporal cost, thanks to the fact that *j << n*. Equation (18) defines a variant of the QSPCM that we will call QSPCM-*j*.

Obviously, QSPCM*-j* increases the total measurement time and the information to be stored in the PDD. However, in Section 4, we show how very low values of *j* are enough to improve uncertainty in the measurements and reduce the maximum errors.

### 3.2. Fast Quasi Single-Point Calibration Method

The total time needed to estimate *R* can still be reduced by applying the methods described in Reference [33], called fast calibration methods I and II (FCM I and FCM II). In essence, the FCM I aims to reduce the discharge time through the highest resistances. To achieve this, the discharge through *R* stops after a preset time *T_x_* (*T_x_* > *T_Rc1_*), followed immediately by the discharge of the capacitor through *R_c1_* until completion. This discharge procedure will be referred to as the *R* accelerates the discharge procedure. There is therefore a reduction in the measurement times of all resistors with *T_R_* > *T_x_*, and this reduction increases as the value of *R* increases. Using the FCM I, the value that *T_R_* would have with the normal discharge procedure can be found, according to the expression:

(19) $$T_{R}=\frac{T_{Rc1}}{T_{Rc1}-T_{Rc1}^{\prime }(R)}T_{x},\;T_{R}\geq T_{x}$$

where $T_{Rc1}^{\prime }(R)$ is the time discharging through *R_c1_* after discharging through *R* for time *T_x_*. If we call the time used in the accelerated discharge procedure $T_{R}^{*}$, this can be calculated using the following expression:

(20) $$T_{R}^{*}=T_{x}+T_{Rc1}^{\prime }(R)$$

Let Δ*T_R_* = Δ*T_R_(R,R_c1_,T_x_)* be the difference in measurement times between the normal discharge procedure and the accelerated discharge procedure for *R*. Its value is therefore given by:

(21) $$\begin{array}{c} \Delta T_{R}=\Delta T_{R}\left(R,R_{c1},T_{x}\right)=T_{R}-T_{R}^{*}=T_{R}-\left(T_{x}+T_{Rc1}^{\prime }(R)\right)=T_{R}-\left(T_{x}+\frac{T_{R}-T_{x}}{T_{R}}\cdot T_{Rc1}\right)=\\ =\left(T_{R}-T_{x}\right)\left(1-\frac{T_{Rc1}}{T_{R}}\right) \end{array}$$

Equation (21) shows that, obviously, the reduction in measurement time increases as *T_x_* decreases. However, the choice of *T_x_* also has implications for the maximum error in estimating *R*. Thus, the smaller *T_x_* (and also, in consequence, the time needed to find *T_R_*), the greater the error in estimating *R* may be, although Reference [33] shows that there may be an optimal *T_x_* values zone where this phenomenon does not occur.

For its part, in the FCM II, the accelerated discharge procedure applies to both *R* and *R_c2_*, with *T_E_*(*R*) decreasing even more. This method will be used to reduce *T_E_*(*R*), even though it also comes at a small cost in terms of accuracy of results. Obviously, *T_Rc1_* < *T_x_* < *T_Rc2_* must occur in order to apply the method.

In the FCM II, using Equation (19) for both *R* and *R_c2_*, the estimation of *R* is given by:

(22) $$R=\{\begin{array}{cc} \frac{T_{R}-T_{Rc1}}{\frac{T_{Rc1}}{T_{Rc1}-T_{Rc1}^{\prime }(R_{c2})}T_{x}-T_{Rc1}}\left(R_{c2}-R_{c1}\right)+R_{c1}, & T_{R}\leq T_{x};\;\;\;T_{Rc1}<T_{x}<T_{Rc2}\\ \frac{\frac{T_{x}}{T_{Rc1}-T_{Rc1}^{\prime }(R)}-1}{\frac{T_{x}}{T_{Rc1}-T_{Rc1}^{\prime }(R_{c2})}-1}\left(R_{c2}-R_{c1}\right)+R_{c1}, & T_{R}>T_{x};\;\;\;T_{Rc1}<T_{x}<T_{Rc2} \end{array}$$

Using $T_{R}^{n}$ and $T_{Rc2}^{0}$ calculated from the measurements made by the accelerated discharge procedure, we obtain a new calibration method determined by the equations equivalent to Equations (14) and (15). This method, which we will call Fast-QSPCM, is defined by:

(23) $$R^{n}=\{\begin{array}{cc} \frac{T_{R}^{n}-T_{Rc1}^{n}}{T_{Rc1}^{n}}A^{*}\left(R_{c2}-R_{c1}\right)+R_{c1}, & T_{R}^{n}\leq T_{x};\;\;\;T_{Rc1}<T_{x}<T_{Rc2}\\ \left(\frac{T_{x}}{T_{Rc1}^{n}-T_{Rc1}^{n{}^{\prime }}(R^{n})}-1\right)A^{*}\left(R_{c2}-R_{c1}\right)+R_{c1}, & T_{R}^{n}>T_{x};\;\;\;T_{Rc1}<T_{x}<T_{Rc2} \end{array}$$

where $T_{Rc1}^{n{}^{\prime }}(R^{n})$ is the time for which the capacitor is discharged through *R_c1_* after having done so through *R* in the *n*^th^ estimation. For its part, A^*^ is described as:

(24) $$A^{*}=\frac{1}{\frac{T_{x}}{T_{Rc1}^{0}-T_{Rc1}^{0{}^{\prime }}(R_{c2})}-1}$$

Operating in the same way with Equations (17) and (18), the equation that defines another calibration method, Fast-QSPCM-*j*, is obtained:

(25) $$R^{n}=\{\begin{array}{cc} \frac{T_{R}^{n}-T_{Rc1}^{n}}{T_{Rc1}^{n}}A^{*j}\left(R_{c2}-R_{c1}\right)+R_{c1}, & T_{R}^{n}\leq T_{x};\;\;\;T_{Rc1}<T_{x}<T_{Rc2};\;\;\;1<j\ll n\\ \left(\frac{T_{x}}{T_{Rc1}^{n}-T_{Rc1}^{n{}^{\prime }}(R^{n})}-1\right)A^{*j}\left(R_{c2}-R_{c1}\right)+R_{c1}, & T_{R}^{n}>T_{x};\;\;\;T_{Rc1}<T_{x}<T_{Rc2};\;\;1<j\ll n \end{array}$$

where A^*j^ is the same as *A* defined in Equation (17) but is with *T_Rc2_* of each estimation calculated from the measurements of the accelerated discharge procedure, similarly to that in Equation (24).

In order to compare *T_E_(R)* in the new methods to those obtained in Equations (8) and (9), we are going to use the mean values of *T_E_(R)* (*R* being constant) in estimating *n* resistive sensors (or *n* estimations of the same sensor), $\mu \left(T_{E}(R)\right)$. In the case of the SPCM and the TPCM: $\mu \left(T_{E}(R),SPCM\right)$ and $\mu \left(T_{E}(R),TPCM\right)$ match the values of *T_E_(R)* in Equations (8) and (9), since *T_E_(R)* is the same in any estimation of *R^n^*. However, in the QSPCM and in the Fast-QSPCM, the values of *T_E_(R)* differ in the first estimation. For its part, in QSPCM*-j* and Fast-QSPCM-*j*, the first *j* estimations have a different *T_E_(R)* value from those of the others. Table 1 shows the $\mu \left(T_{E}(R)\right)$ for each method when *n* estimations of *R* are made.

## 4. Experimental Results and Discussion

The performances obtained with the QSPCM*s*, SPCM, and TPCM have been compared using an FPGA (Xilinx XC3S50AN-4TQG144C, Xilinx Inc., San Jose, CA, USA) [34] as a PDD mounted on a FR-4 fiberglass substrate with four layers. The FPGA uses a quartz crystal to generate a 50 MHz operating frequency and needs two regulators (TPS79912 and TPS79633, Texas Instruments, Dallas, TX, USA) to power the core of the device at 1.2 V and the I/O buffers at 3.3 V. This limits the noise that digital activity can generate on the device’s input and output pins. The I/O buffers have been programmed to provide the maximum current allowed by the manufacturer (i.e., 24 mA) in order to maintain digital integrity of the signals. The resistance measurement results are transmitted via an SPI to a controller and finally sent to a PC via a USB flash drive.

A series of 20 resistors with values between 267.56 Ω and 7464.5 Ω was used in order to evaluate the performances of the different methods, as these values are within the range of a large number of resistive sensors and, in particular, tactile sensors. Selecting a 47 nF capacitor ensured the discharge times for all these resistors were measured using a 14-bit counter implemented in the FPGA, and, moreover, that the relative maximum error using any method never exceeded 3%. Besides, three additional resistors of 8170 Ω, 9056.1 Ω, and 9963.7 Ω were used solely for the Fast-QSPCM and Fast-QSPCM-*j* methods, since these methods allow the discharge time of these resistors to be measured with the 14-bit counter.

Apart from these resistors, *R_c0_* = 3486.8 Ω was used as a calibration resistor for the SPCM, and *R_c1_* = 1098.1 Ω and *R_c2_* = 6165.3 Ω were used for the other methods. All the resistors were measured using an Agilent 34401A digital multimeter. A number of 500 measurement cycles were performed for each of the 23 resistors used in order to measure the maximum errors and uncertainty. These 500 cycles were repeated again each time the resistors are estimated using a different method. In each cycle, the discharge time was measured through the resistor to be estimated and through one or both calibration resistors, depending on the method used and the measurement cycle in question. Thus, for example, for the QSPCM, discharge is via *R_c2_* only in the first measurement cycle, while in the remaining 499 cycles discharge is only through *R_c1_* and *R*.

**Figure 2:**
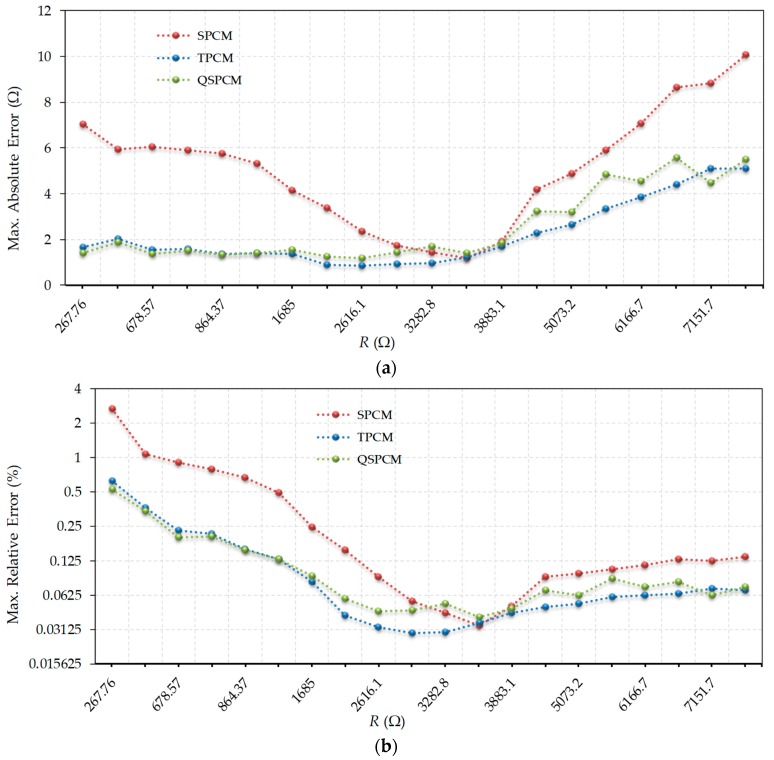
Errors in estimating resistance values using SPCM, TPCM, and QSPCM: (**a**) absolute maximum errors (linear scale); (**b**) relative maximum errors (log2 scale).

Figure 2a shows the maximum errors obtained for the SPCM, the TPCM, and the QSPCM, while Figure 2b shows these same errors but expressed as relative to the nominal value of the resistor to be measured. For easier viewing, it should be noted that the results are presented in a linear scale on the y-axis in Figure 2a, and in a log2 scale for Figure 2b. As expected, the biggest errors always occur in the SPCM, except in the vicinity of its calibration resistor, 3486.8 Ω. Only in this case, Equation (2) is a very good approximation to obtain *R*. The absolute error curve of this method shows a typical “V” shape. For their part, the TPCM and the QSPCM maintain very similar errors to each other throughout the entire range. Absolute errors are practically constant in the low resistance value zone, while absolute errors increase slowly, and relative errors remain practically constant for high resistance values. Especially striking is the low resistance values region, where the SPCM shows relative errors up to 6 times greater than those of the other methods (of which errors are practically identical).

**Figure 3:**
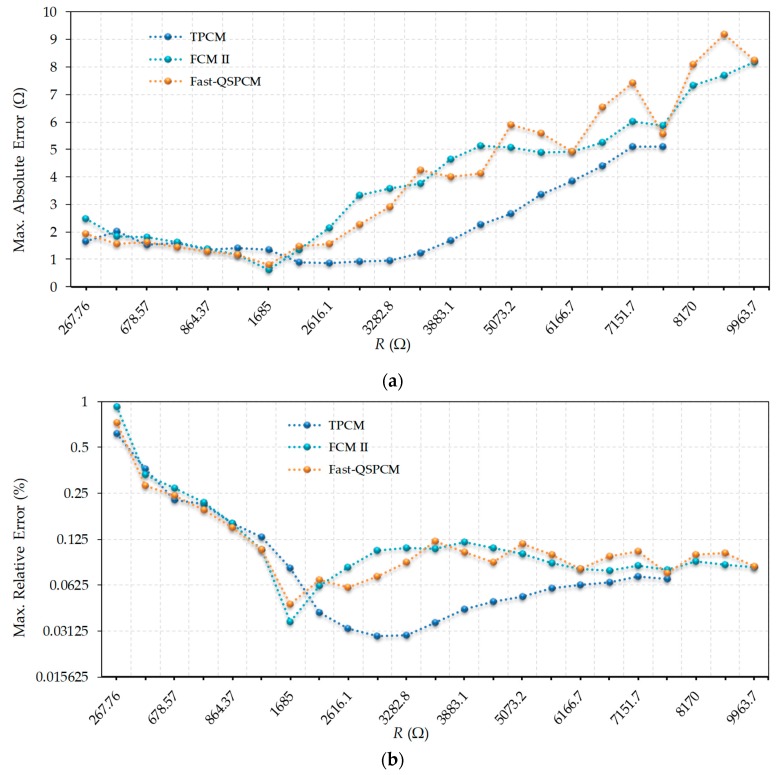
Errors in estimating resistance values using the TPCM, FCM II, and Fast-QSPCM: (**a**) absolute maximum errors (linear scale); (**b**) relative maximum errors (log2 scale).

In order to evaluate the maximum errors in the Fast-QSPCM, Figure 3a compares these errors with those of the TPCM and FCM II. *T_x_* = 163.84 µs was chosen for the comparison, i.e., half the time that can be measured with the 14-bit counter. This value was chosen as it is suitable for monitoring the value of the most significant bit of the counter, in order to know if time *T_x_* was reached during the discharge, thus facilitating the hardware to be designed in the FPGA. This choice implies that all resistors with values under approximately 4000 Ω discharge the capacitor by themselves, while larger resistors use the accelerated discharge procedure to do so. However, as shown in Figure 3a, resistors with values below 4000 Ω do not present the same errors using TPCM and FCM II since, in this second method, *R_c2_* is also evaluated using *R_c1_*. Although this reduces *T_E_(R)*, it also increases the error. For its part, the Fast-QSPCM in Figure 3a presents very similar errors to FCM II, and only shows to some degree greater errors than FCM II for some of the higher resistance values. It is again important to note that the TPCM can be used to measure a maximum resistance value of slightly more than 7500 Ω; however, thanks to the decrease in discharge times for large resistors, the FCM II and the Fast-QSPCM can be used to measure resistors of up to 10 kΩ. For this reason, the graphs in Figure 3 only show the results obtained with FCM II and Fast-QSPCM for resistors with values greater than 7500 Ω. Figure 3b shows the relative errors made by these methods, again in a log2 scale, where it is observed that the relative errors remain practically constant for large resistance values, with very close values in all three methods.

**Figure 4:**
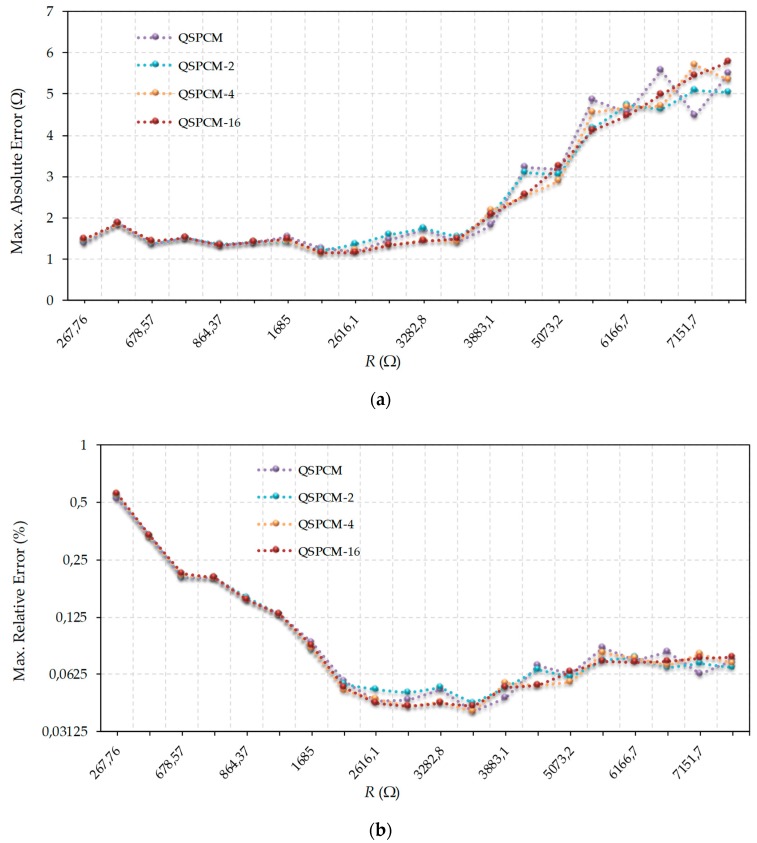
Comparison of errors made in estimating resistance values using the QSPCM and different values of *j* in QSPCM-*j*: (**a**) absolute maximum errors (linear scale); (**b**) relative maximum errors (log2 scale).

Figure 4 compares the results obtained for QSPCM and QSPCM-*j* with j∈{2,4,16}. As expected, the shapes of the curves for the absolute (Figure 4a) and relative (Figure 4b) errors become gentler as *j* increases. However, there does not seem to be any truly significant improvements for *j* > 2, meaning *j* = *2* appears to be a good compromise in terms of error reduction, smoothness of the curve, and hardware complexity.

**Figure 5:**
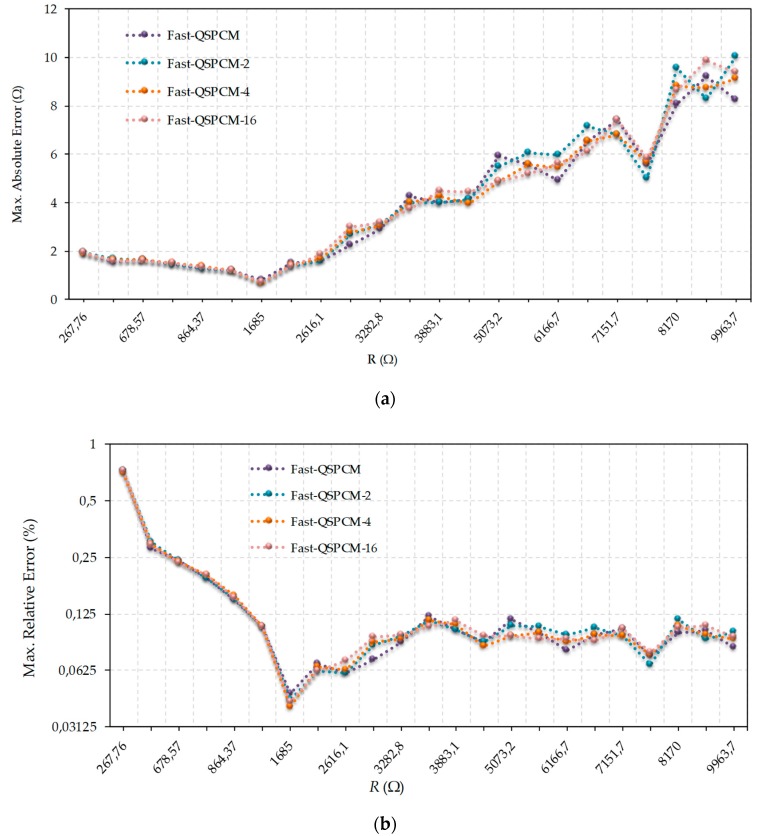
Comparison of errors made in estimating resistance values using Fast-QSPCM and different values of *j* in Fast-QSPCM-*j*: (**a**) absolute maximum errors (linear scale); (**b**) relative maximum errors (log2 scale).

The results shown in Figure 5 are similar to the previous ones for Fast-QSPCM and Fast-QSPCM-*j* with j∈{2,4,16}. Again, an increase in *j* translates into smoother error curves for absolute (Figure 5a) and relative (Figure 5b) errors. However, these graphs show slightly better results than the rest for *j* = 4 and would seem to be the best choice. In fact, neither Figure 4 nor Figure 5 shows major differences between the results for the different methods. It is the designer who should select the most suitable method for each application.

To round off this section, Table 2 shows a summary of the performances of the methods analyzed when estimating a range of resistors with *R_max_* = 7464.5 Ω and *R_min_* = 267.56 Ω. The results are calculated based on data used for Figure 2, Figure 3, Figure 4 and Figure 5 and equations listed in Table 1. The *T_x_* = 163.84 µs was retained for the Fast-QSPCM and Fast-QSPCM*-4* methods.

The second and third columns of Table 2 show the maximum errors obtained with any resistor within the range used. The penultimate column of the Table 2 shows the time needed to estimate *R_max_* according to the different methods when estimating a single sensor once. For its part, the last column shows the case in which 20 estimations are made (for a single sensor or 20 different sensors). Particularly striking is the fact that the Fast-QSPCM reduces the mean time for 20 estimations by 61% compared to the TPCM, or by 47% compared to the SPCM. However, the maximum relative error only increases by 0.1% compared to that with the TPCM and is 3.65 times lower than that with the SPCM. For their part, the QSPCM and the QSPCM-2 present the lowest relative errors of all the methods, and yet show important reductions in the mean estimation times compared to the SPCM and the TPCM.

## 5. Conclusions

The TPCM is a calibration method commonly used in DICs for measuring resistive sensors, since it presents fewer errors in estimations than the SPCM. However, the TPCM needs more time to perform the estimation as it has to measure discharge times for three resistors: the resistor to be estimated and two calibration resistors.

Although the FCM has recently been proposed in order to reduce estimation time, this reduction may not be enough when having to make repetitive measurements of the values of a sensor or when having to measure the values of a large number of sensors. For these situations, this paper proposes a series of new methods, QSPCMs, in which two calibration resistors are also used but one of them is only measured in an initial estimation. Some of the proposed methods are based on the same discharge procedures as the TPCM, while others, in order to reduce the mean estimation time, use accelerated discharge procedures, as the FCM.

In order to compare the new proposed methods with traditional methods, a circuit with an FPGA (Xilinx XC3S50AN-4TQG144C) has been used to measure resistances values in the range (267.56 Ω, 7464.5 Ω). When 20 estimates of the range’s maximum resistance value are made, one of the proposed methods, the Fast-QSPCM achieves a reduction of 61% in the mean estimation time compared to the TPCM, while the relative maximum error for any resistance value in the range only increases by 0.1%.

## Figures and Tables

**Table 1:** Mean time for an estimation of *R*, if *n* estimations are made, both in traditional methods and in the methods presented in this paper.

Method	$\mathrm{Mean}\;\mathrm{of}\;T_{E}R\;\mathrm{for}\;n\;\mathrm{Estimations},\;\mu \left(T_{E}(R)\right)$
SPCM	$T_{R}+T_{Rc0}$
TPCM	$T_{R}+T_{R\max}+T_{R\min}$
Fast calibration method II (FCM II)	$\left\{ \begin{array}{c} \mu \left(T_{E}(R,TPCM)\right)-\Delta T_{R}\left(R,R_{c1},T_{x}\right),\;T_{R}>T_{x}\\ \mu \left(T_{E}(R,TPCM)\right),\;T_{R}\leq T_{x} \end{array}\right.$
Quasi single-point calibration method (QSPCM)	$T_{R}+T_{Rc1}+\frac{T_{Rc2}}{n}$
Fast single-point calibration method (Fast-QSPCM)	$\left\{ \begin{array}{c} \mu \left(T_{E}(R,QSPCM)\right)-\Delta T_{R}\left(R,R_{c1},T_{x}\right)-\frac{\Delta T_{R}\left(R_{c2},R_{c1},T_{x}\right)}{n},\;T_{R}>T_{x}\\ \mu \left(T_{E}(R,QSPCM)\right),\;T_{R}\leq T_{x} \end{array}\right.$
QSPCM*-j*	$T_{R}+T_{Rc1}+j\frac{T_{Rc2}}{n}$
Fast-QSPCM-*j*	$\left\{ \begin{array}{c} \mu \left(T_{E}(R,QSPCM-j)\right)-\Delta T_{R}\left(R,R_{c1},T_{x}\right)-j\frac{\Delta T_{R}\left(R_{c2},R_{c1},T_{x}\right)}{n},\;T_{R}>T_{x}\\ \mu \left(T_{E}(R,QSPCM-j)\right),\;T_{R}\leq T_{x} \end{array}\right.$

**Table 2:** Comparison of the performance of the different methods for a range of resistors with values between 267.56 Ω and 7464.5 Ω.

Method	Max. Absolute Error (Ω)	Max. Relative Error (%)	$\mu \left(T_{E}(R_{\max})\right)$ µs	
			1 estimation	20 estimations
SPCM	10.07	2.63	449.83	449.83
TPCM	5.11	0.62	605.00	605.00
FCM II	6.00	0.92	409.11	409.11
QSPCM	5.56	0.52	605.00	364.54
Fast-QSPCM (*T_x_* = 163.84 µs)	7.42	0.72	409.11	238.36
QSPCM*-2*	5.10	0.54	605.00	377.20
Fast-QSPCM-4 (*T_x_* = 163.84 µs)	6.79	0.71	409.11	265.32
